# The effect of ketamine on psychopathology and implications for understanding schizophrenia and its therapeutic use: a meta-analysis

**DOI:** 10.1192/bjo.2021.634

**Published:** 2021-06-18

**Authors:** Katherine Beck, Guy Hindley, Faith Borgan, Cedric Ginestet, Robert McCutcheon, Stefan Brugger, Naomi Driesen, Mohini Ranganathan, Deepak D'Souza, Matthew Taylor, John Krystal, Oliver Howes

**Affiliations:** 1 IoPPN King's College London; 2 Yale University School, Dept. of Psychiatry and National Centre for PTSD, VA Connecticut; 3 University Department of Psychiatry, Warneford Hospital

## Abstract

**Aims:**

To conduct a meta-analysis of the effect of ketamine on psychopathology in healthy volunteers and patients with schizophrenia, and the experimental factors affecting this.

**Background:**

Ketamine is increasingly used to treat depression and other psychiatric disorders but can induce schizophrenia-like symptoms. Despite this, the consistency and magnitude of symptoms induced by ketamine, or what factors influence the effects of ketamine on these remain unknown.

**Method:**

MEDLINE, EMBASE and PsychINFO databases were searched for within-subject placebo controlled studies reporting symptoms using the Brief Psychiatric Rating Scale (BPRS) or Positive and Negative Syndrome Scale (PANSS) in response to an acute ketamine challenge in healthy participants or people with schizophrenia. Two independent investigators extracted study-level data for a random-effects meta-analysis. Total, positive and negative BPRS and PANSS scores were extracted. Sub-group analyses were conducted examining the effect of: blinding status, ketamine preparation, infusion method and time between ketamine and placebo condition. Standardized mean change scores were used as effect sizes for individual studies. Standardized mean changes between ketamine and placebo for total, positive and negative BPRS and PANSS were calculated.

**Result:**

Of 7819 citations retrieved, 36 studies involving healthy participants were included. The overall sample included 725 healthy volunteers exposed to both the ketamine and placebo condition. Ketamine induced a significant increase in transient psychopathology in healthy participants, for total (Standardized mean change (SMC) = 1.50 (95% CI = 1.23 to 1.77), p < 0.0001), positive (SMC = 1.55 (95% CI = 1.29 to 1.81), p < 0.0001) and negative (SMC = 1.16, (95% CI = 0.96 to 1.35), p < 0.0001) symptom ratings, relative to the placebo condition. This effect was significantly greater for positive symptoms than negative symptoms (p = 0.004). Bolus followed by constant infusion increased ketamine's effect on positive symptoms relative to infusion alone (p = 0.006). Single-day study design increased ketamine's effect on total symptoms (p = 0.007), but age and gender did not moderate effects. There were insufficient studies for meta-analysis of studies in schizophrenia. Of these studies, two found a significant increase in symptoms with ketamine administration in total and positive symptoms. Only one study found an increase in negative symptom severity with ketamine.

**Conclusion:**

These findings show that acute ketamine administration induces schizophrenia-like symptomatology with large effect sizes but there is a greater increase in positive than negative symptoms, and when a bolus is used. These findings suggest bolus doses should be avoided in its therapeutic use to minimize the risk of inducing transient positive psychotic symptoms.

